# Dosimetric evaluation of an intraoperative radiotherapy system: a measurement-based and Monte-Carlo modelling investigation

**DOI:** 10.1007/s13246-023-01243-6

**Published:** 2023-03-23

**Authors:** Marsha Chin, Pejman Rowshanfarzad, Gabor Neveri, Martin A. Ebert, David Pfefferlé

**Affiliations:** 1 School of Physics, Mathematics and Computing, University of Western Australia, 35 Stirling Highway, Mailbag M013, CRAWLEY, Perth, WA 6009 Australia; 2 Department of Radiation Oncology, Sir Charles Gairdner Hospital, Nedlands, WA Australia

**Keywords:** Intraoperative radiotherapy, Intrabeam, Monte Carlo, Dosimetry

## Abstract

Intraoperative radiotherapy (IORT) is a specialised subset of radiotherapy, where a high radiation dose is delivered to a surgically exposed tumour bed in order to eradicate any remaining cancer cells. The aim of this study was to examine the dose characteristics of the Zeiss Intrabeam IORT device which provides near-isotropic emission of up to 50 kV X-rays. The EGSnrc Monte Carlo (MC) code system was used to simulate the device and percentage depth dose (PDD) data measured with a soft X-ray parallel-plate ionisation chamber were used for model verification. The model provided energy spectra, isodose curves and mean photon energies. In addition, EBT3 Gafchromic film was used to verify the MC model by examining PDDs and 2D dose distributions for various applicators. The differences between MC model and ionisation chamber measurements were within 3% for most points, with a maximum deviation of ~ 9%. Most of the simulated PDD points were within 5% of the film-measured data, with a maximum deviation of ~ 10%. The mean energy of the bare probe was found to be 21.19 keV. The mean photon energy from applicators ranged from 29.00 to 30.85 keV. Results of this study may be useful for future work on creating a system for treatment planning.

## Introduction

Intraoperative radiotherapy (IORT) involves the precise delivery of a high dose of radiation to a surgically exposed tumour or tumour bed, with healthy tissues either shielded or displaced out of the radiation field. The dose is delivered in a single fraction, and usually between 10 and 20 Gy [1].

The Zeiss Intrabeam system is a low-kV IORT device delivering near-isotropic X-rays. A cathode produces electrons that are accelerated down a probe towards a gold target. The electron energy (and consequently maximum X-ray energy) can be set at 30, 40 or 50 kV with a current of 5, 10, 20 or 40 µA [2]. Zeiss offers treatment versatility via different types of Intrabeam applicators: spherical, needle, flat and surface. Spherical applicators can be used for intracavitary IORT, *e.g.,* during breast conserving surgery.

EGSnrc is a Monte Carlo (MC) software package used to model the transport of ionisation radiation through matter. EGSnrc is capable of simulating photons, electrons and positrons with kinetic energies from 1 keV to 10 GeV in homogeneous media [3]–elements, compounds or mixtures. A C + + library allows for the design of complex simulation geometries and particle sources. EGSnrc also includes the BEAMnrc software component, which in turn includes DOSXYZnrc–a dose-scoring utility which allows the estimation of radiation dose in a rectilinear voxel geometry. Further data processing tools enable a detailed analysis of beam characteristics as well as the generation of radiation dose curves [4].

The manufacturer-recommended method for dosimetry of the Intrabeam system involves the Zeiss water tank and a soft X-ray parallel-plate ionisation chamber. Despite being the gold standard, ionisation chambers only provide one-dimensional dose data. Film measurements are valuable especially because they offer high resolution two-dimensional dose data, *i.e.,* dose distribution across a plane. However, there are few articles on Intrabeam dosimetry with film measurements, and even fewer which use the film directly in water [5, 6].

With access to a laser cutter, the film-cutting process can be automated, allowing film to be cut with greater precision and reproducibility than with scissors [7]. The slow water penetration rate of Gafchromic films in general make it feasible to perform measurements directly in water [8]. With access to a 3D printer, water-equivalent structures can be printed which allow for accurate positioning of film in water.

At the time of writing, the study by Watson et al. [9] was the only report in literature with film measurements performed directly in water using a 3D-printed holder. They investigated depth doses along the same axis as the Intrabeam probe but made no measurements with applicators.

There have been several studies on the simulation of the Intrabeam source using various MC software toolkits (GATE, GEANT4, MCNP, MCDS, EGSnrc) [6, 10–18], but only a few have used EGSnrc, and very few simulate the spherical applicators in addition to the bare probe. Only Nwankwo et al*.* [14] and Alvarez et al*.* [10] included all three coatings of the Intrabeam probe (NiO, Ni and CrN). The coatings have a distinct impact on the resulting energy spectra. Therefore, it is desirable to have a more accurate MC simulated model including these details which many studies are missing.

This study aims to create a Monte Carlo model for the Zeiss Intrabeam system, not only for the bare probe but also for the spherical applicators, and to verify the MC model using an ionisation chamber as well as Gafchromic film measurements in water.

## Methods

### Zeiss intrabeam

The Intrabeam core system contains the PRS 500 control console and XRS 4 X-ray source. The control console supplies a low DC voltage to the X-ray source, which generates a high voltage to direct the electron beam into the probe [19]. The X-ray generator body (7 cm × 11 cm × 11 cm) attaches to a floor stand which allows for six degrees of freedom to treat various sites of a patient’s body [2].

This study involved the Intrabeam bare probe and spherical applicators, with diameters ranging from 1.5 cm to 5 cm in 0.5 cm increments. The XRS4 X-ray source was set to 50 kV.

### Monte Carlo simulations

**Fig. 1:**
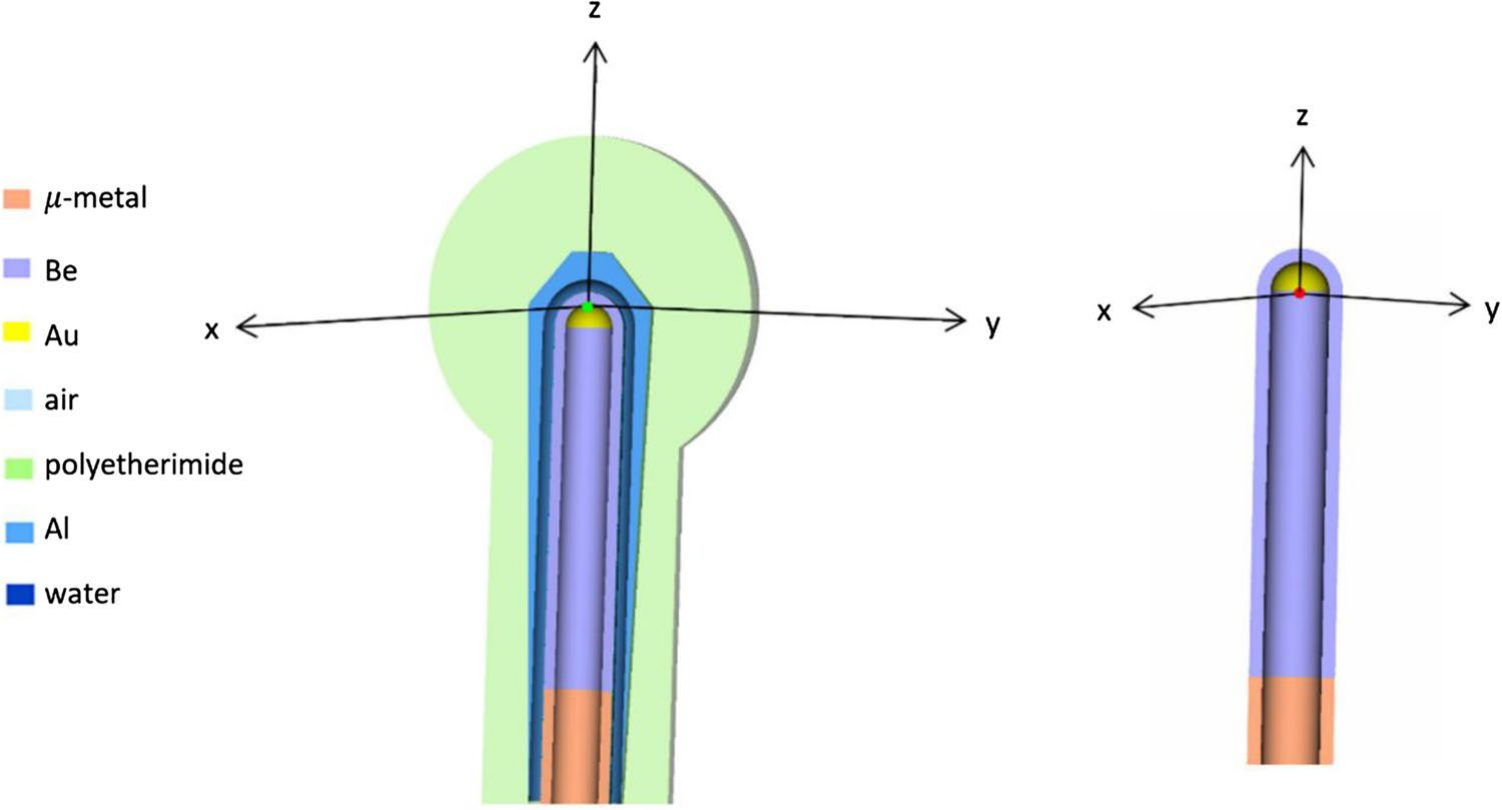
x-, y- and z-directions for the Intrabeam applicator and probe. The origin for the applicator models (green dot) and for the bare probe model (red dot) are also shown. (Color figure online)

The 2021 release version of EGSnrc [20] was used for MC simulations. The *x*-, *y*- and *z*-directions defined for MC models are shown in Fig. 1. For the applicator models, the origin was located at the applicator isocentre (green dot). For the bare probe, the origin was at the centre of the gold hemisphere (red dot), corresponding to the probe isocentre. Simulations were performed in two steps:

1. Modelling of the bare probe to obtain a phase space file, with photons scored as they left the probe
2. Modelling of spherical applicators using the phase space file developed in Step 1 to calculate dose

Table 1 shows the materials that make up the Intrabeam probe. Electrons are accelerated down a 10-cm-long probe, 3.2 mm in diameter, towards a 1-µm-thick gold target [14]. The probe is an evacuated cylindrical tube, made of µ-metal except for a 1.6-cm-long beryllium exit window at the tip. The electrons strike the target, producing bremsstrahlung photons in an approximately isotropic manner.

**Table 1:** Intrabeam probe materials and composition [10, 16]

		Material	Thickness (µm)
Target		Au	1
Body	Proximal 8.4 cm	$\mu$-metal	500
	Distal 1.6 cm	Be	500
Biocompatible coatings		NiO	2.5
		Ni	2.5
		CrN	2.5

The electron source was modelled based on the findings of Clausen et al*.* (2012) [12]—the electron beam strikes the gold target with two rings: 0.6 mm to 0.7 mm in radii (weighting factor of 1.05) and 0.7 mm to 0.8 mm in radii (weighting factor of 1.55). This source has a Gaussian energy distribution with a mean energy of 50 keV and full width half maximum (FWHM) of 5 keV, in accordance with vendor specification [12].

Eight applicators were simulated, with diameters of 1.5 cm to 5 cm in increments of 0.5 cm. Each applicator consisted of a ‘shank’ and a ‘ball’ – both had dimensions that were unique to the applicator. The shank was composed of a wider cylindrical section, a conical section, and a thinner cylindrical section which attached to the ball. For applicators with diameters ≤ 3 cm, there was a thin aluminium layer in the applicator to attenuate low-energy photons [16]. Applicator dimensions were not provided by the manufacturer and therefore, planar X-ray images were taken of all applicators to determine the geometries for modelling.

**Fig. 2:**
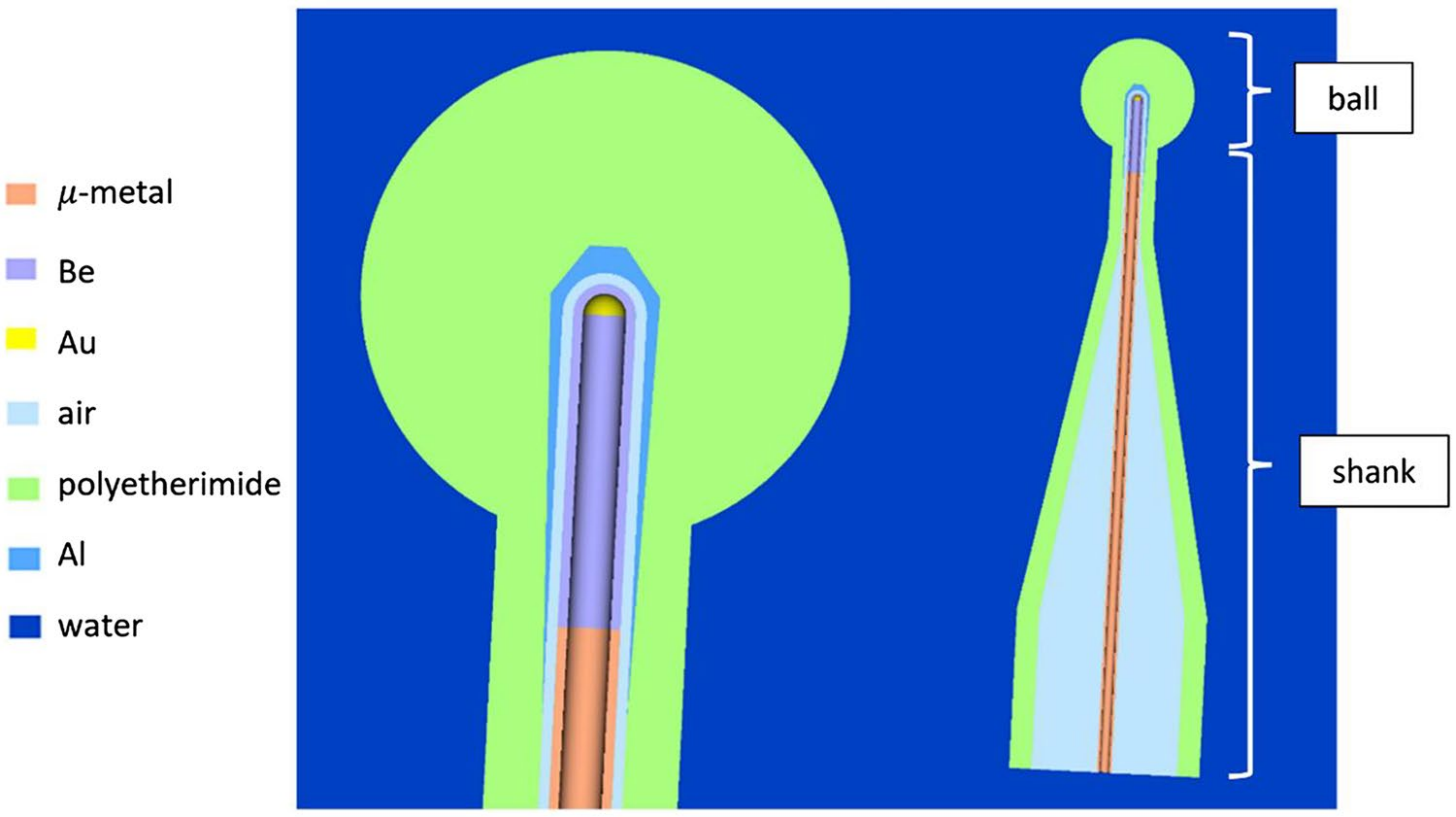
Cross-sectional illustration of the MC model for the 2.5-cm-diameter applicator in EGSnrc. The probe is inserted into the air gap inside the applicator. The exterior probe coatings (NiO, Ni, CrN) are too thin to be visualised

The spherical applicators were made of a biocompatible polyetherimide thermoplastic (GE ULTEM 1000) [16], C_37_H_24_O_6_N_2_, with a density of 1.27 g/cm^3^ [19]. Within the applicators was a hollow region of air; this was where the probe was inserted. Again, applicators with diameters ≤ 3 cm had an aluminium layer on the interior which acted as an attenuator, removing characteristic photons and the low-energy tail of X-rays [19]. Figure 2 shows the MC design of the 2.5-cm-diameter applicator.

The applicator and probe geometries were simulated in a water phantom (30 cm × 20 cm × 30 cm) that was divided into voxels of 0.16 cm × 0.16 cm × 0.1 cm. The 0.16 cm width in the *x*- and *y*-dimensions matched the sensitive volume of the ionisation chamber, and the resolution of 0.1 cm was used to yield dose information in the steep fall-off region near the probe/applicator in the *z*-direction. However, to extract the MC PDD in the *x*-direction, the voxel width was adjusted to 0.1 cm to get more data points. The dose in each voxel and associated uncertainty was recorded in a *.3ddose* file and analysed using Python.

Phase-space files with at least 10^9^ particles were generated. Each subsequent bare probe and applicator simulation was calculated with 10^9^ histories. The maximum statistical uncertainty was 6.5% (a coverage factor of *k* = 3). The minimum and maximum thresholds for photon production were 1 keV and 200 keV, respectively. The electron energy threshold for absorption was set to 513 keV, which is the total energy of the electron, including the rest mass energy of 511 keV. Therefore, all electrons with less than 2 keV kinetic energy were absorbed in their current region. The photon energy threshold for absorption was set to 1 keV. Simulations were performed on an Intel(R) Xeon(R) CPU E5-2640 v2 @ 2.00 GHz with 32 cores and 132 GB.

To fit a curve to the MC PDD data for each probe/applicator, two equations were used:

1 $$y = \,\frac{{100Ae^{ - Bx} }}{{x^{2} }} + \,D$$

2 $$y = \,100Ae^{ - Bx} + \,100Ce^{ - Dx}$$

Equation 1 was used for the applicators. The $1/{x}^{2}$ term represents the loss due to inverse square law, and the decaying exponential term represents the attenuation in material. Equation 2 was used for the bare probe, as it provided a better fit.

For ease in curve fitting, Eq. 1 was rearranged to be of the form

3 $$y = \,100Ae^{{ - Bx\, - \,2{\text{In}}\,x\, + \,\,C}}$$

prior to curve fitting.

### Ionisation chamber measurements

Measurements were made in a Zeiss Intrabeam Water Phantom (580 mm × 400 mm × 520 mm) using a PTW 34013 soft X-ray parallel-plate ionisation chamber (PTW, Freiburg, Germany) in conjunction with a PTW UNIDOS E electrometer (PTW, Freiburg, Germany). The ionisation chamber was not waterproof and was therefore inserted into a (fixed) built-in waterproof holder inside the water tank. The Zeiss water tank positioning system allowed the probe/applicator to be adjusted horizontally and vertically.

Measurements were made with the bare probe and all spherical applicators. Six to seven depths were measured for each probe/applicator, and three readings were taken at each depth. Micrometre screws on the Zeiss positioning system allowed for positioning within $\pm$ 0.1 mm [21]. The Zeiss dosimetry protocol [22] was used to calculate the dose rate at each distance. These dose rates were normalised and plotted against distance from the probe tip to produce PDD curves.

### Zeiss dosimetry protocol

Based on the Zeiss dosimetry protocol [22], the absorbed dose rate to water (${\dot{D}}_{w}(r)$) at a distance *r* is calculated from Eq. 4:

4 $$\mathop {D_{w} }\limits^{ \cdot } \left( r \right) = \,N_{k} \cdot \,Q\left( r \right) \cdot \,\frac{T}{{T_{0} }} \cdot \,\frac{{P_{0} }}{P} \cdot \,k_{Q} \cdot k_{{k_{{A^{{ \to D_{w} }} }} }}$$

where *N*_*k*_ is the detector calibration factor (Gy/C), *Q(r)* is the charge (C) measured over a defined time interval, *T* is the current temperature (K), *T*_*0*_ is the reference temperature (K), *P*_*0*_ is the reference air pressure (hPa), *P* is the current air pressure (hPa), *k*_*Q*_ is the beam quality correction factor, and ${k}_{{k}_{A}\to {D}_{w}}$ is the correction factor for air kerma to absorbed dose to water conversion for the PTW 34013 ionisation chamber.

*T*_*0*_, *P*_*0*_ and *k*_*Q*_ were obtained from the ionisation chamber calibration certificate supplied by Zeiss [23]. The Zeiss dosimetry protocol [22] states that the quality level of the Intrabeam source is approximately equivalent to a T30 reference beam (HVL = 0.37 mm Al), *i.e.*, *k*_*Q*_ = 1. The PTW laboratory is traceable to the Physikalisch-Technische Bundesanstalt (PTB), the national standard of the German National Laboratory which supplied the three chamber correction factors (*N*_*k*_, *k*_*Q*_ and ${k}_{{k}_{A}\to {D}_{w}}$) [24]. The measured room temperature *T* and pressure *P* were used to correct for deviations from the reference conditions.

### Uncertainty analysis

The estimation and propagation of uncertainties were based on the ‘Guide to the expression of uncertainty in measurement’ (GUM) by the International Organisation of Standardization (ISO) [9, 24, 25]. The uncertainty in dose rate (${\dot{D}}_{w}(r)$), ${\sigma }_{Zeiss}$, was calculated using Eq. 5:

5 $$\sigma_{Zeiss} = \,\sqrt {\sigma_{rep}^{2} + \,\sigma_{pos}^{2} + \sigma_{{k_Q}k_{k_A \to D_{W}}}^{2} + \,\sigma_{P}^{2} + \,\sigma_{T}^{2} }$$

where ${\sigma }_{rep}$ was the standard deviation of the mean of ionisation chamber readings, ${\sigma }_{pos}$ was the relative uncertainty in chamber positioning, ${\sigma }_{{k}_{Q}{k}_{{k}_{A}\to {D}_{w}}}$ was the relative uncertainty from the product of ${N}_{k}$, ${k}_{Q}$ and ${k}_{{k}_{A}\to {D}_{w}}$, and ${\sigma }_{P}$ and ${\sigma }_{T}$ were the uncertainties in pressure and temperature measurements.

### Film measurements

#### Film holder

**Fig. 3:**
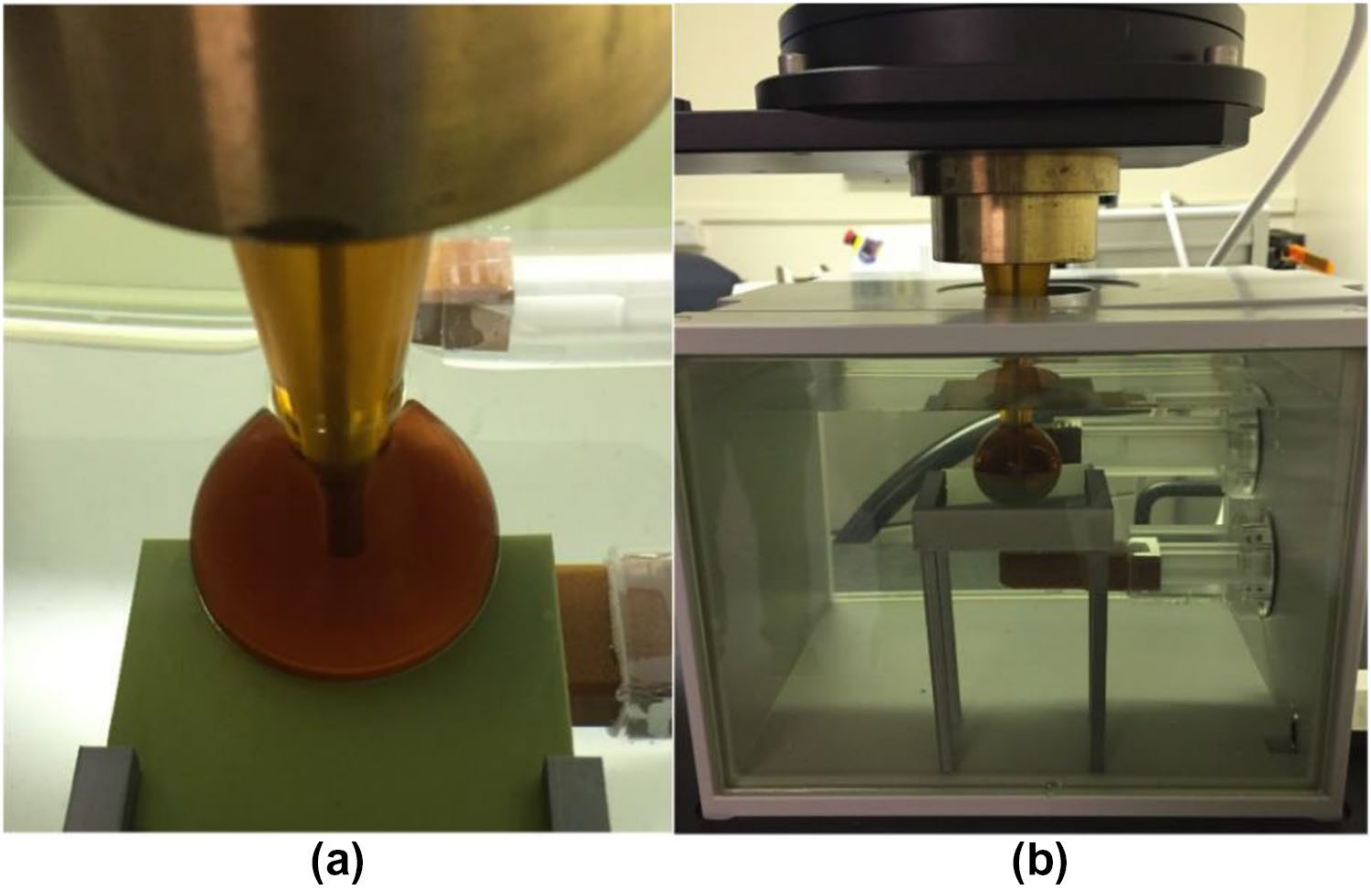
Experimental set-up for the film irradiation. **a** Close-up view of the film-applicator positioning. **b** Film table and film immersed in the Zeiss water tank

The film holder was designed and 3D-printed in-house. It consisted of two parts: the tabletop and the legs (Fig. 3(b)). Both parts were designed using the Fusion 360® software (Autodesk, California, USA). A narrow slit allowed the film to slide through (tabletop), and cuboidal cut-outs allowed for the insertion of the 8-cm legs. The film table and leg designs were exported as STL files and imported into Prusa Slicer software (Prusa Research, Prague, Czech Republic). Prusa printers (Prusa Research, Prague, Czech Republic) were used to 3D-print the film holder components.

#### Film slices

Films were cut in two ways. The first set were 90 mm × 60 mm, with a hemisphere cut into one side. The hemisphere varied in size, ranging from the bare probe to the 4.5 cm applicator diameter. These film slices were designed on Fusion 360 software and imported into the laser cutter control system. The second set of films which were used for measurements with the bare probe, were 60 mm × 60 mm pieces with a cut-out circle of radius 1.6075 mm (same as the probe).

#### Film irradiations

Films were irradiated with an energy setting of 50 kV on the Intrabeam, with the goal of obtaining dose data in the *xy*-plane (Fig. 1). The films were placed in the film holder, which was submerged in the Zeiss water tank. Using a laser cutter to cut the film perhaps melted and sealed the edges of the film as there was no visible water damage around the edges. Film irradiations were performed for the 1.5, 2.5, 3.5 and 4.5 cm applicators. Three films were irradiated per applicator.

**Fig. 4:**
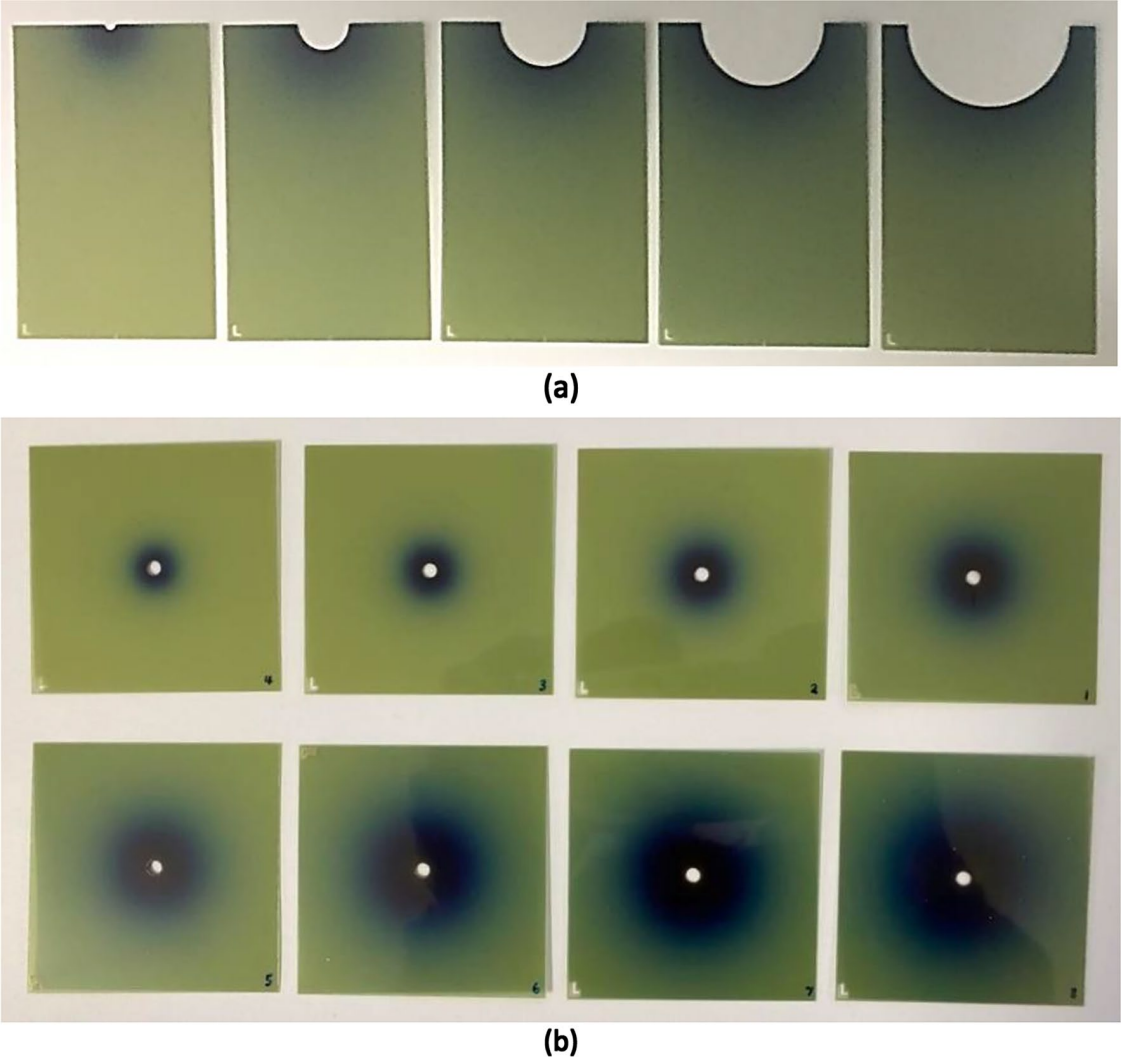
**a** Irradiated films using: the bare probe, 1.5 cm, 2.5 cm, 3.5 cm and 4.5 cm applicators, and **b** irradiated film using the bare probe with delivered doses of 0.25, 0.5, 1, 2, 4, 8, 12 and 16 Gy at 10 mm from the probe centre

For each set-up, 2 Gy was delivered at 10 mm from the probe or applicator surface. Figure 3 shows the experimental set-up for film exposures using an applicator. To ensure that the film was pressed right against the applicator, the film table was positioned against the wall of the water tank, and the Zeiss positioning system was used to push the applicator against the film. Care was taken to ensure there were no air bubbles on the film or elsewhere. The irradiated films were immediately dried after exposure by placing them on soft tissue paper. They were then left in a black envelope in a box for 24 h before being scanned. Figure 4a shows some of the irradiated films.

The films for the bare probe were irradiated with an energy setting of 50 kV on the Intrabeam, with the same set-up as above. Each film was positioned at the *z* = 0 plane, corresponding to a height of approximately 13 mm on the Zeiss positioning frame. Each film was irradiated with different doses: 0.25, 0.5, 1, 2, 4, 8, 12 and 16 Gy at 10 mm from the probe centre (red dot in Fig. 1). Figure 4b shows the irradiated films.

#### Film scanning and analysis

The film was scanned using an Epson Expression 12000XL scanner, with 48 bit and 300 dpi settings. To obtain PDDs, the red channel of the image was used, as this is the most sensitive channel for dosimetry for doses < 2 Gy [26]. Pixel values were extracted along the central *x*-axis using MATLAB R2022a. To minimise noise and improve accuracy, the central five rows of pixels along the *x*-axis were averaged, a median filter was used on the average set, and the resulting values were used to create the PDD curve.

All of the scanned films were analysed using MATLAB and the isodose curves were plotted for each applicator. It must be noted that that the films were positioned in the *z* = 0 plane, not at the tip of the applicator or probe.

#### Uncertainty analysis

In this section, a film ‘set’ refers to film slices belonging to the same probe/applicator.

To determine the uncertainty associated with every pixel value:

1. For each film in each film set, the standard deviation across the five rows of pixel data was determined. This produced an overall uncertainty vector (uncertainty at each depth), ${\sigma }_{slic{e}_{i}}$, for that particular film slice *i*. There were three slices of film irradiated per probe/applicator, producing three independent ${\sigma }_{slic{e}_{i}}$ vectors where $i\, \epsilon \left\{\mathrm{1,2},3\right\}.$
2. Then, for each corresponding depth in ${{\sigma}_{slice_1}}$, ${{\sigma }_{slice_2}}$ and ${{\sigma }_{slice_3}}$, the standard deviation was determined. This gave an overall uncertainty vector for each probe/applicator film measurement, ${\sigma }_{film}$, *i.e.,* a vector containing uncertainty at each pixel (or depth) along the film.

## Results

Calculation of each phase space file required an average of about 24 h, and the applicator simulations required about 9 h each (only one core was used per simulation).

### PDD curves

#### Comparison of MC simulations with ionisation chamber measurements (*z*-axis)

**Fig. 5:**
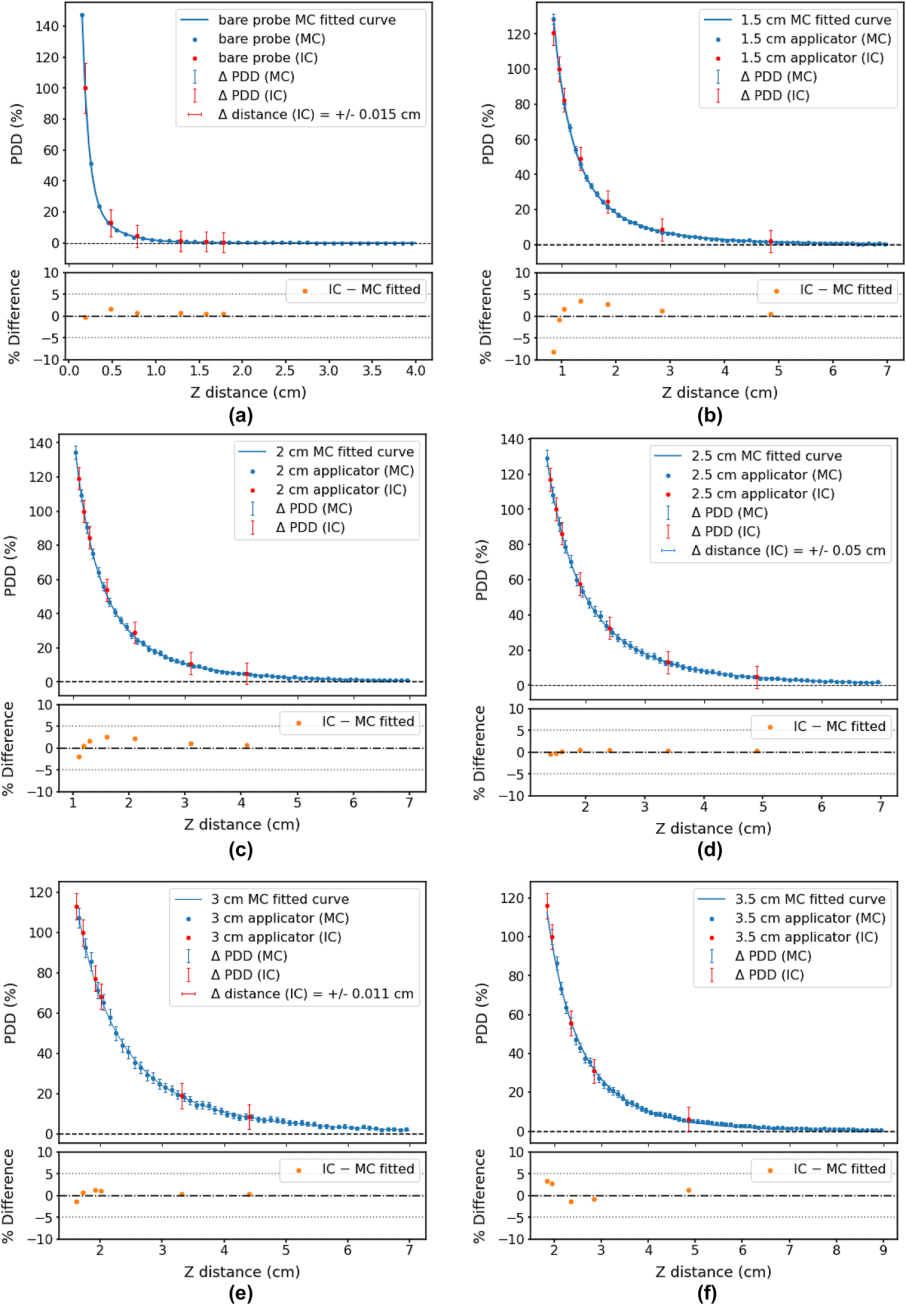

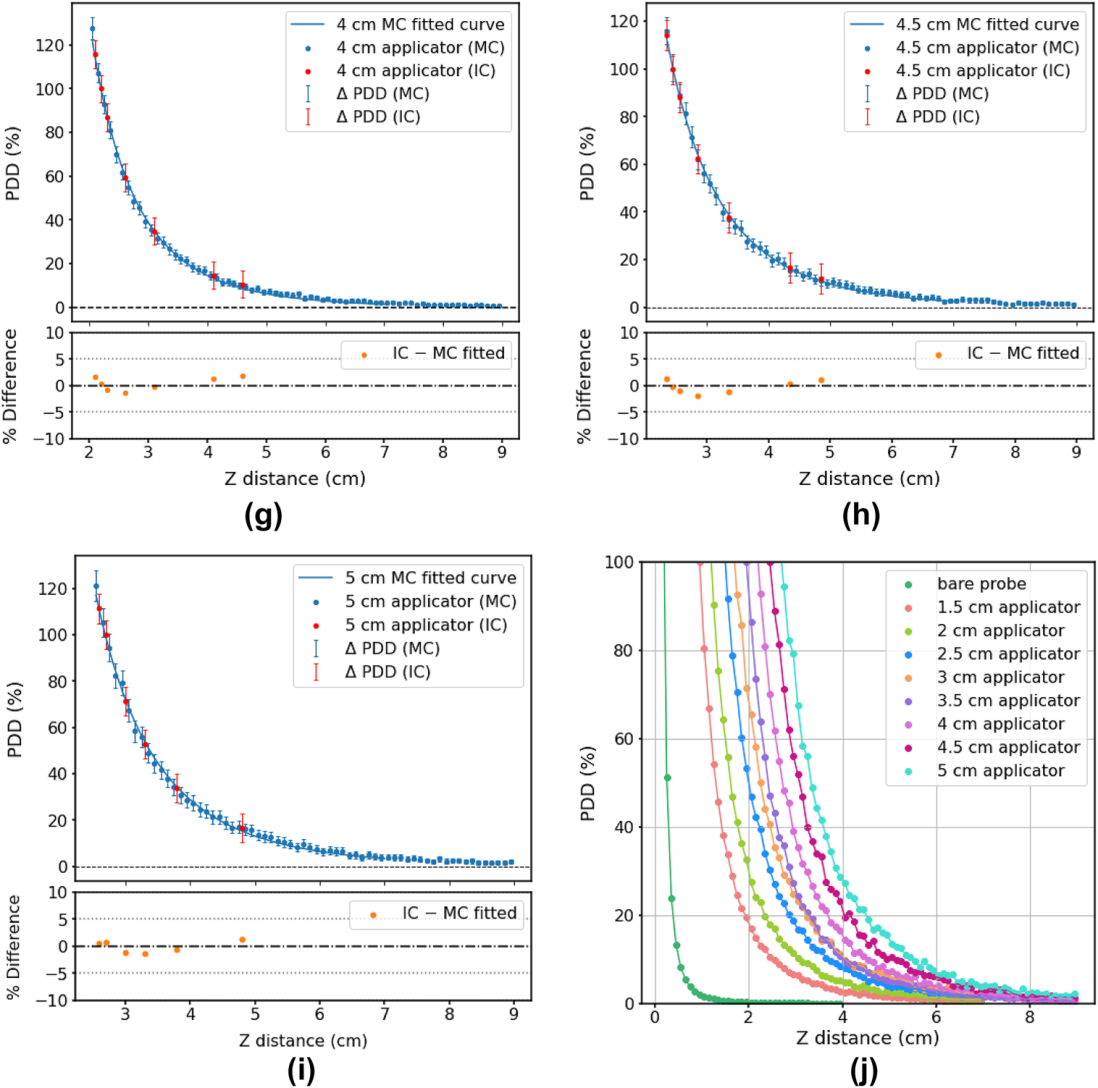
Comparison of PDD curves from MC simulations and ionisation chamber (denoted by IC) measurements for the bare probe and spherical applicators of diameters 1.5, 2, 2.5, 3, 3.5, 4, 4.5 and 5 cm, with the percentage differences between MC (fitted curve) and ionisation chamber data points shown for each. All error bars had a coverage factor of *k* = 3 (99.7% confidence interval). The MC PDDs for the bare probe and all applicators are included in (**j**)

MC-simulated PDDs along the *z*-axis were generated for the bare probe and each of the spherical applicators. These results were verified against ionisation chamber measurements along the same axis. Figure 5 shows a comparison of MC and ionisation chamber results, with a plot of percentage differences below the PDDs. All error bars were given a coverage factor of *k* = 3 (99.7%). Table 2 shows the fitted parameters for the MC curves according to Eqs. 2 and 3.

**Table 2:** Fitted parameters for the MC PDD curves (*z*-axis) according to Eq. 2 (bare probe) and Eq. 3 (applicators)

Applicator/probe	A	B	C	D	R^2^
probe	9.163	13.845	0.542	3.460	0.9999
1.5 cm app	0.8	0.22	0.335	–	0.9999
2.0 cm app	1.009	0.221	0.613	–	0.9997
2.5 cm app	0.674	0.222	1.539	–	0.9998
3.0 cm app	1.183	0.223	1.279	–	0.9989
3.5 cm app	1.732	0.403	1.544	–	0.9980
4.0 cm app	2.889	0.404	1.401	–	0.9977
4.5 cm app	1.431	0.352	2.297	–	0.9981
5.0 cm app	2.560	0.344	1.968	–	0.9975

The R^2^ values are also given

The bare probe PDD was normalised to the closest ionisation chamber depth, 0.1992 cm from the probe isocentre. Applicator PDDs were normalised to a depth of 0.2 to 0.25 cm from the applicator surface, depending on the available ionisation chamber points.

For all probe and applicator measurements, at all depths measured, ${\sigma }_{rep}$ was less than 0.41%. ${\sigma }_{pos}$ was due to a $\pm$ 0.01 mm uncertainty in the positioning unit of the water tank and was converted to a relative uncertainty for each depth measurement. The calibration certificate of the ionisation chamber reported ${\sigma }_{{k}_{Q}{k}_{{k}_{A}\to {D}_{w}}}$= 2% for a coverage factor of *k* = 1 [9, 23, 24]. ${\sigma }_{P}$ was $\pm$ 0.01 hPA which was negligibly small when converted to a relative error. ${\sigma }_{T}$ was $\pm {0.1}^{\circ }C$ and was less than 0.48% for all measurements. As a result, the mean value for ${\sigma }_{Zeiss}$ was 2.32% for all individual measurements.

#### Comparison of MC simulations with film measurements (*x*-axis)

**Fig. 6:**
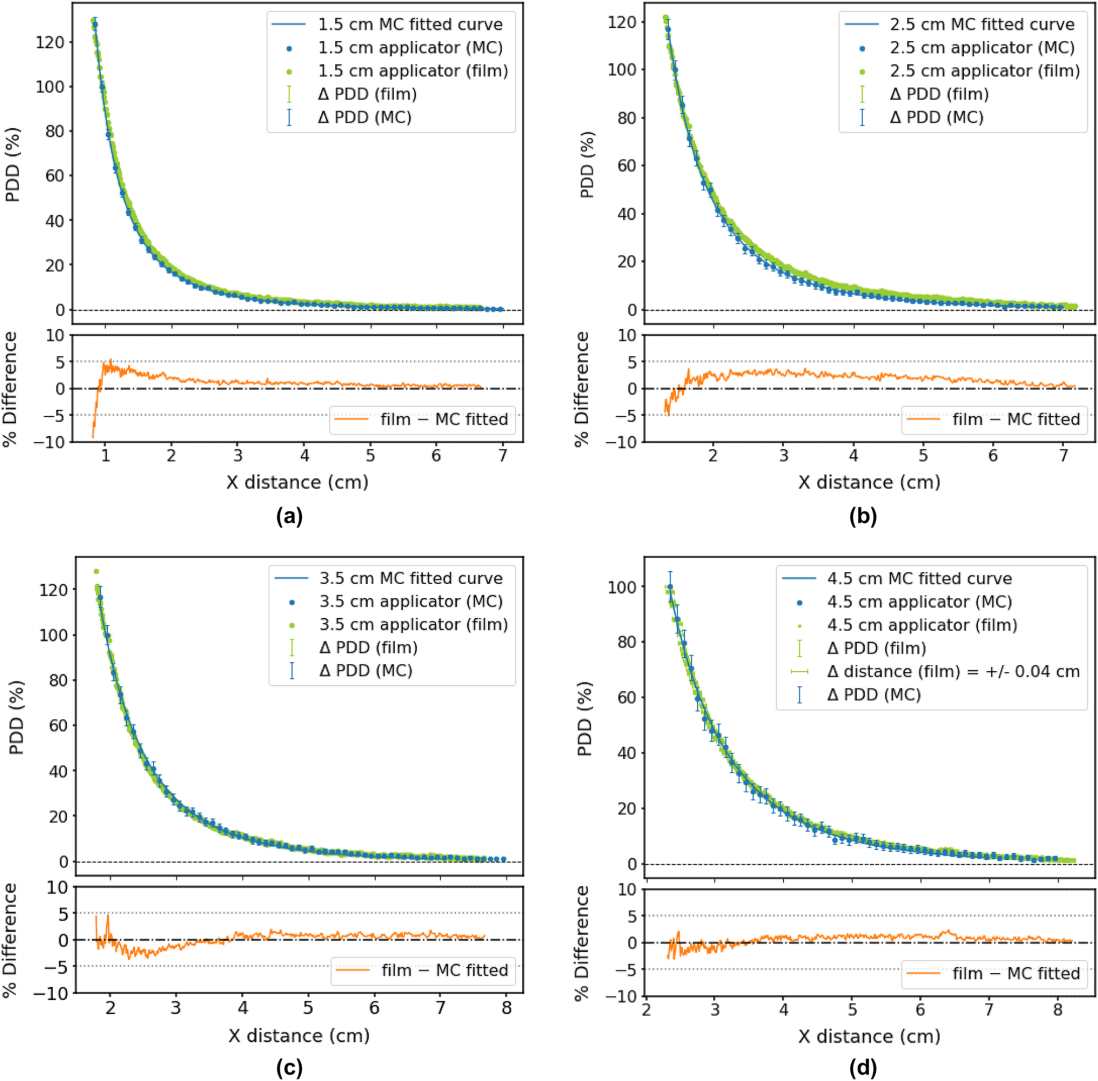
Comparison of PDD curves from MC simulations and film measurements in *x*-axis for spherical applicators of diameters 1.5, 2.5, 3.5 and 4.5 cm, with the percentage differences between MC (fitted curve) and film data points shown for each. All error bars had a 99.7% confidence interval

Figure 6 shows the PDD curves in the *x*-direction, comparing MC and film data for four applicators (1.5, 2.5, 3.5 and 4.5 cm). Vertical error bars for film measurements were derived from the deviation between the central five rows of pixels on film scans. They were, in general, too small and not visible on Fig. 6 (< 0.12%). The film PDD for the 4.5 cm applicator was shifted by 0.04 cm horizontally to produce better agreement (Fig. 6d). This was possibly due to uncertainty in film positioning and seemed reasonable since the uncertainty associated with the distance setting was estimated to be ± 0.1 cm. Table 3 shows the fitted parameters for the MC curves according to Eq. 3.

**Table 3:** Fitted parameters for the MC PDD curves (*x*-axis) according to Eq. 3 (applicators)

Applicator	A	B	C	R^2^
1.5 cm app	0.706	0.272	0.497	0.9998
2.5 cm app	1.875	0.266	0.492	0.9995
3.5 cm app	1.870	0.392	1.456	0.9983
4.5 cm app	2.743	0.356	1.519	0.9976

The R^2^ values are also given

#### Energy spectra

**Fig. 7:**
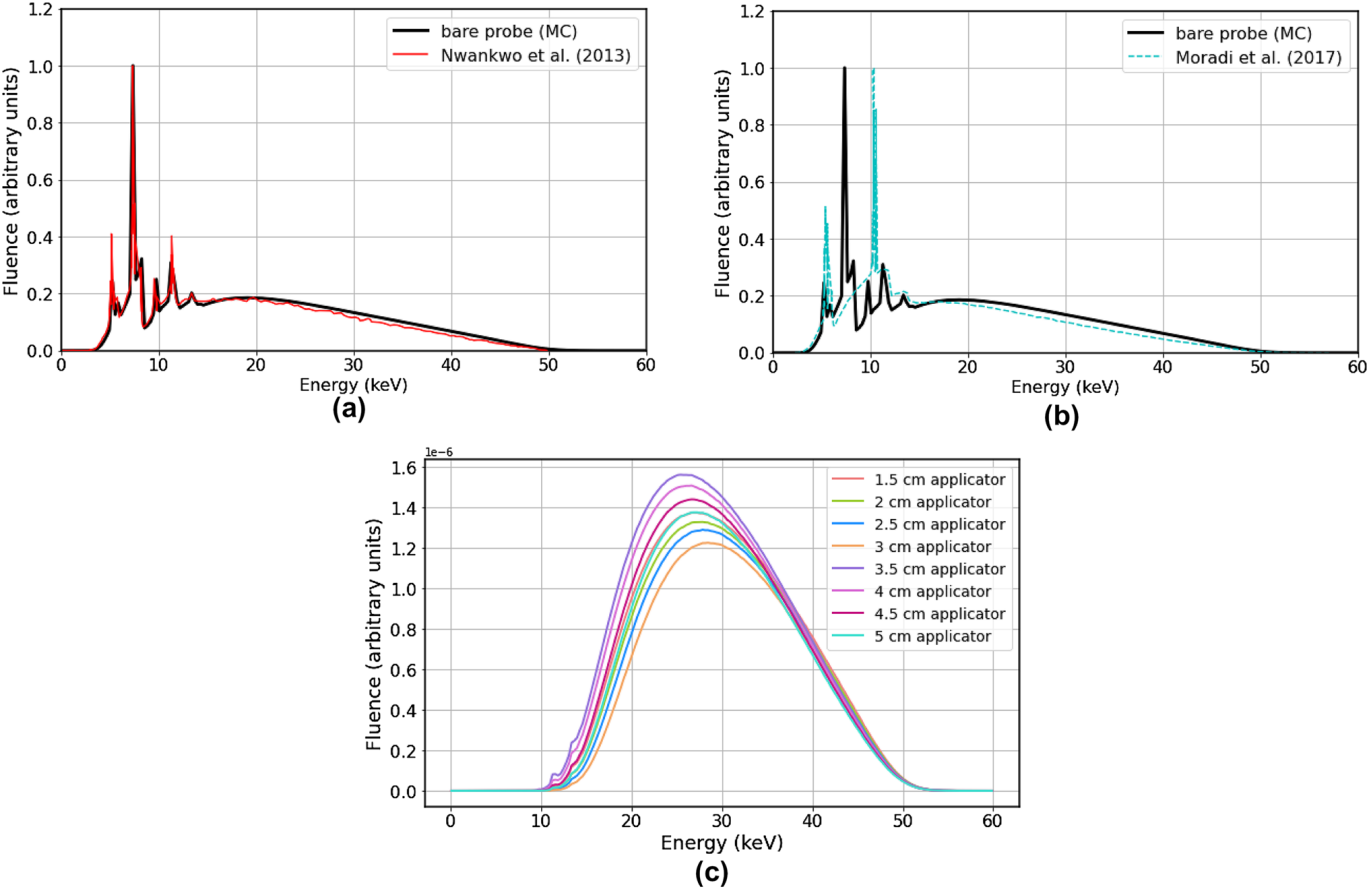
Energy spectrum of the 50 kV Intrabeam bare probe and applicators from Monte Carlo simulations with probe geometry including three coatings (NiO, Ni and CrN) and comparison to: **a** Nwankwo et al. (2013) [14], which does include the three coatings, and **b** Moradi et al. [6], which only includes the CrN coating. **c** Comparison of energy spectra for all spherical applicators, scored at the applicator surface (note: only applicators with diameters ≤ 3 cm had an aluminium attenuator)

Figure 7a shows the energy spectrum for the 50 kV bare probe at the probe surface. The data reported by Nwankwo et al*.* [14] are also plotted for comparison. It must be noted that the work by Nwankwo et al*.* is one of the two studies to date that include all three coatings of the Intrabeam probe—NiO, Ni and CrN. Results of another study which had only used the CrN coating (Moradi et al*.* [6]) are shown in Fig. 7b to emphasise the impact of MC geometry design on the energy spectrum. Figure 7c shows the energy spectrum for all spherical applicators using a 50 kV energy setting.

The average energy for each probe/applicator is listed in Table 4. All spectra are extracted at the applicator surface.

**Table 4:** Mean X-ray energy for various Intrabeam set-ups (probe and all applicators) extracted from MC simulations

Probe/applicator	Mean energy (keV)
Probe	21.19
1.5 cm app	30.04
2 cm app	30.29
2.5 cm app	30.51
3 cm app	30.85
3.5 cm app	29.00
4 cm app	29.20
4.5 cm app	29.52
5 cm app	29.80

The Intrabeam source was modelled with a Gaussian energy distribution with maximum energy 50 keV and FWHM of 5 keV

#### Isodose curves

**Fig. 8:**
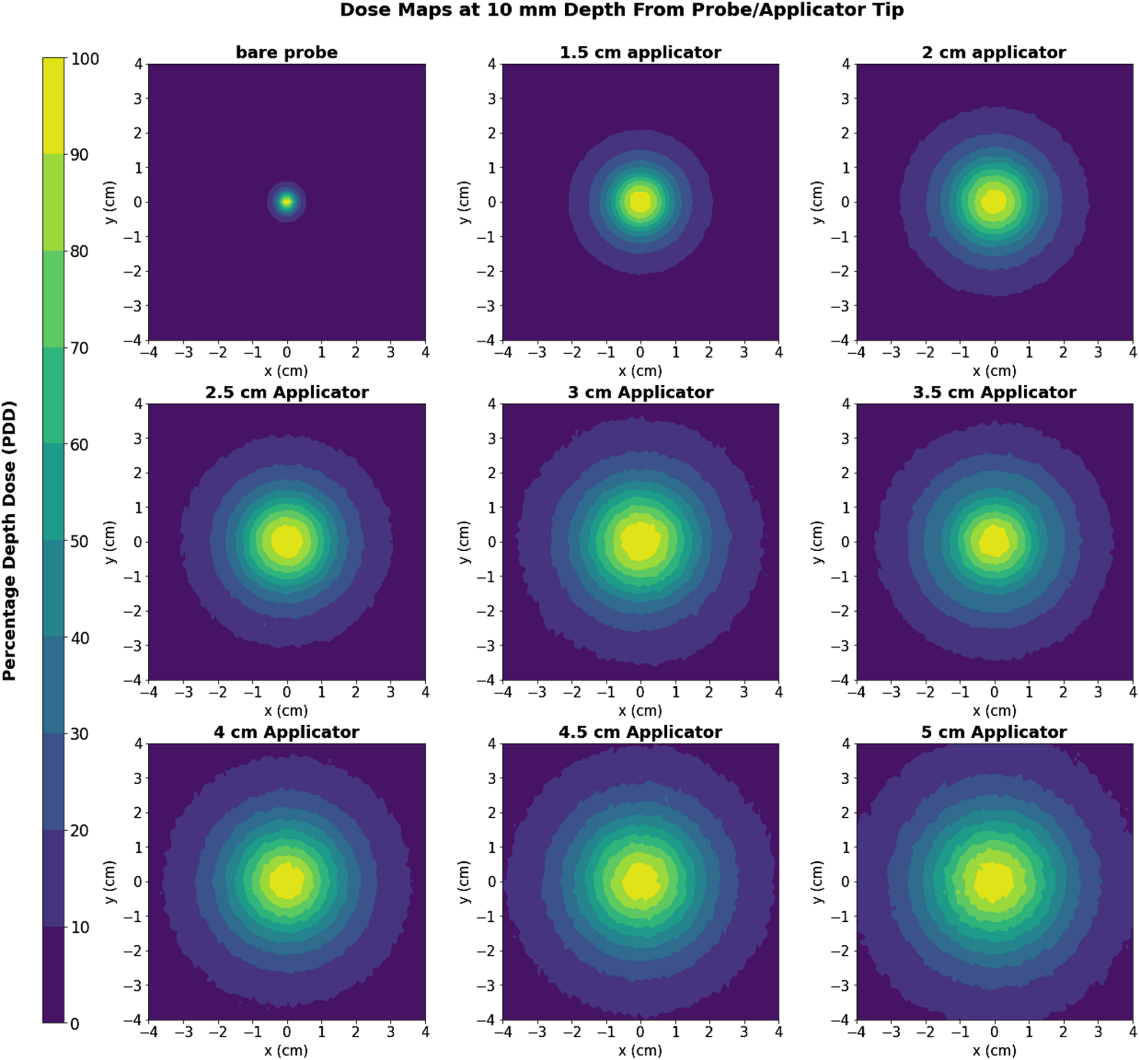
MC simulated isodose curves for the bare probe and spherical applicators, in the *xy-*plane along the central *z*-axis at a depth of 10 mm from the probe/applicator surface

The isodose curves extracted from MC simulations of the bare probe and spherical applicators are shown in Fig. 8. The plot axes represent the *xy*-plane along the central *z*-axis at a depth of 1 cm from the probe or applicator surface. Isodose curves are drawn at 0 to 100% dose regions in 10% intervals, as indicated by the colour bar on Fig. 8.

**Fig. 9:**
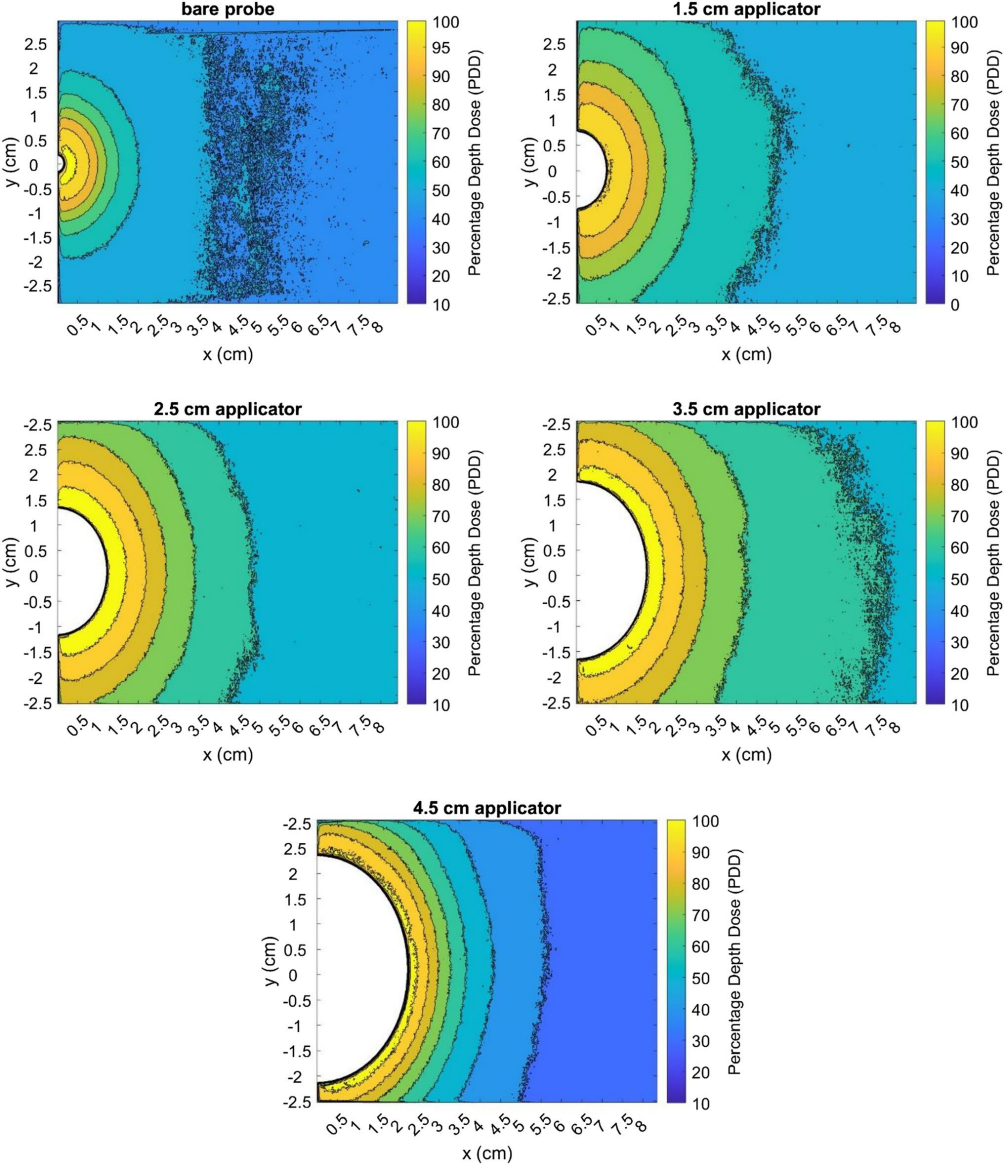
Film measured isodose curves for the bare probe and spherical applicators (1.5, 2.5, 3.5 and 4.5 cm), in the *xy*-plane along the central *z*-axis at a depth of *z* = 0, *i.e.* the central plane of the applicator balls and the beginning of the probe hemisphere

The isodose curves from film measurements were also extracted. Figure 9 shows the isodose curves for the bare probe, and 1.5, 2.5, 3.5, and 4.5 cm applicators. They were all irradiated with a 50 kV energy setting. The applicator films were prescribed with 2 Gy at 10 mm from the applicator surface. The probe was prescribed with 2 Gy at 10 mm from the centre.

## Discussion

### Percentage depth doses (PDDs)

#### Comparison of MC simulations with ionisation chamber measurements (*z*-axis)

Figure 5 illustrates a comparison between MC-simulated and ionisation chamber-measured PDDs along the central axis of the bare probe, and for different spherical applicators. Most MC-simulated and ionisation chamber-measured values agreed within 3%. The exception was the 1.5 cm applicator, which showed a maximum deviation of about 9%. The bare probe, and the 2.5 cm and 3 cm applicator curves exhibited a clear systematic shift between MC and ionisation chamber data. For this reason, the ionisation chamber data were shifted horizontally by 0.015 cm, 0.05 cm and 0.011 cm respectively. Such manipulations of data were indicated on the PDD plots as horizontal error bars. These systematic shifts were most likely due to uncertainty in the ionisation chamber positioning, or the probe/applicator positioning. The latter was controlled by the Zeiss positioning system, which had an uncertainty of ± 0.01 mm according to the manufacturer [27].

With MC uncertainties of up to 6.48% (coverage factor of *k* = 3), it would be desirable to repeat simulations using more histories to reduce the uncertainty, since it is proportional to 1/$\sqrt{N}$, where *N* is the number of histories. Reducing the MC voxel size, particularly in the *z*-direction, may be beneficial in obtaining a more accurate fitted curve, but this would be unhelpful unless the number of histories were substantially increased to get reasonable dose per voxel. With a small enough voxel size, a curve might not even need to be fitted to MC data—direct comparison between IC and MC PDD values at the same depth may be possible.

Furthermore, it would certainly be worthwhile making ionisation chamber measurements at more depths to allow better comparison to MC data. The normalisation depth for each applicator was slightly inconsistent (± 0.05 cm) due to the limited (ionisation chamber) data points. More ionisation chamber data points would increase both precision and accuracy.

Nonetheless, it is clear from Fig. 5j that the bare probe exhibited the sharpest dose fall-off. For spherical applicators, the maximum dose was at the applicator surface. The applicators allow dose to be deposited fairly isotropically at greater depths, allowing radiotherapy to be better adapted to patients’ needs. The primary reason that applicator dose fall-off gradients are not as steep as the bare probe is the inverse square law. Scoring voxels further away from the probe axis will receive less X-ray fluence, and therefore less dose. The X-ray attenuation by the applicator ball may also contribute to the decreased dose gradient, though this effect is less significant [16]. In general, the smaller the applicator, the steeper radial dose fall-off. Therefore, smaller applicators would allow for greater skin sparing and shorter treatment times [28]. However, it is important to ensure that there are no air gaps present between the applicator and tumour bed.

#### Comparison of MC simulations with film measurements (*x*-axis)

Distal to the source, MC and film measurements agreed within 5%. However, the steep dose fall-off at closer depths mean that small shifts between MC and film plots resulted in a marked difference, almost up to 10% in the case of the 1.5 cm applicator. Each plot exhibited a similar trend in its differences: for smaller distances, the percentage difference was negative, while for greater distances, the percentage difference became slightly positive. Better film calibration could improve the results, or perhaps there was a source of uncertainty affecting only the first few mm of film, such as water damage at the edges or heat damage from the laser.

The PDD curves along the X-axis were very similar to the PDD curves along the Z-axis. This was unsurprising as the dose distribution was expected to be fairly isotropic.

#### Energy spectra

From Figs. 7a and b, it was evident that the coatings of the bare probe made a marked difference in the energy spectrum, which could in turn affect the simulation results. The first set of peaks in Fig. 7a (5.4 and 6 keV) were characteristic X-ray energies for chromium [6]. The greater characteristic X-ray energies (10.4, 11.8 and 13.6 keV) were attributed to the gold target [6]. The largest set of peaks in between corresponded to the characteristic X-ray energies of nickel (7.4 and 8.3 keV) [29].

Results of this study showed the same energy peaks ($\pm$ 0.2 keV) as Nwankwo et al. [14], which included the same materials in the MC probe design. The studies which did not include the nickel oxide and nickel coatings in MC simulations (such as Moradi et al*.* [6]) did not exhibit the same set of energy peaks in the bare probe spectrum, as shown in Fig. 7b.

The energy spectra for the spherical applicators (Fig. 7c) demonstrated a general trend of greater attenuation with larger applicators. However, the aluminium filter in the smaller applicators (1.5 cm to 3 cm) clearly made a difference. The applicator order with the highest fluence peak to the lowest were: 3.5, 4, 4.5, 1.5, 5, 2, 2.5 and 3 cm. The aluminium filter had indeed hardened the beam and shifted the 1.5, 2, 2.5 and 3 cm applicator energy peaks to the right. All applicator balls attenuate the original X-rays generated by the gold target to some extent, eliminating low energy photons. This causes the energy spectra to take on the shape that they have (compared to the original bare probe spectrum).

#### Mean photon energy

The average energy of the bare probe (Table 4) was found to be 21.19 keV. For a 50 kV source, Moradi et al*.* [6] found an average energy of 19.45 keV, which is a difference of 8.21%. In this study, the mean photon energy from applicators ranged from 29.00 to 30.85 keV. Moradi et al. [6] found the mean applicator photon energies to be between 27.8 to 29 keV at the applicator surface. Shamsabadi et al. [16] produced an even lower range: 25.6 to 28.6 keV. This discrepancy could be due to several differences in MC geometry design—perhaps small in impact individually but producing a notable cumulative effect. Both of these studies used a 0.5-µm-thick gold target, as opposed to 1-µm-thick [14]. In addition, they did not include the NiO and Ni coatings of the bare probe. Furthermore, due to the limited information provided by the manufacturer, it is difficult to know the exact applicator dimensions. Nonetheless, the mean photon energies found in this study are reasonable for a 50 kV Intrabeam source.

#### Isodose curves

The MC isodose curves clearly showed steeper radial dose fall-off with the bare probe and smaller applicators, as previously discussed. Results from the irradiated film demonstrated the same effect. A visual inspection of both MC-simulated and film-measured isodose curves shows that X-rays are deposited fairly isotropically, particularly at distances closest to the probe/applicator surface. This agrees with the manufacturer’s descriptions.

It must be noted that the bare probe film isodose curves indicated a slight dose build-up along the *x*-axis. This was not in contradiction to the PDD curve, which showed that the dose was greatest at the probe tip (*z* = 0.16075 cm depth) and decreased with depth, since the film was positioned at *z* = 0 (not the probe tip). This plane corresponded to the beginning of the hemisphere section of the tip, where attenuation and backscatter could have caused the interesting ‘dose build-up’ that was observed.

## Conclusion

Results of this study have direct clinical relevance and may be useful for future work on treatment planning, providing greater insight into dose distribution. The MC phase space file created in this study may act as a virtual source to enable more efficient simulations in the future, whether for further research or treatment planning.

## Data Availability

The datasets generated during the study are available from the corresponding author on reasonable request.
